# Adult Cerebellopontine Angle Medulloblastoma: A Systematic Review of Clinical Features, Management Approaches, and Patient Outcomes

**DOI:** 10.3390/cancers16244242

**Published:** 2024-12-20

**Authors:** Kishore Balasubramanian, Abdurrahman F. Kharbat, Francisco Call-Orellana, Sherwin A. Tavakol, Grace R. Fassina, Christopher Janssen, Othman Bin Alamer, Jeffrey A. Zuccato, Ian F. Dunn

**Affiliations:** 1 College of Medicine, Texas A&M University, 8447 Riverside Parkway, Bryan, TX 77807, USA; kishore-balasubramanian@ouhsc.edu (K.B.); cnjanssen@icloud.com (C.J.); 2 Department of Neurosurgery, University of Oklahoma Health Sciences Center, 1000 N. Lincoln Ave. #4000, Oklahoma City, OK 73104, USA; abdurrahman-kharbat@ouhsc.edu (A.F.K.); sherwin-tavakol@ouhsc.edu (S.A.T.); grace-fassina@ouhsc.edu (G.R.F.); jeffrey.zuccato@ouhealth.com (J.A.Z.); 3 Department of Neurosurgery, The University of Texas MD Anderson Cancer Center, 1400 Holcombe Boulevard, Houston, TX 77030, USA; facall@mdanderson.org; 4 Department of Neurosurgery, Loma Linda University Medical Center, 11234 Anderson St., Loma Linda, CA 92354, USA; obinalamer@llu.edu

**Keywords:** medulloblastoma, cerebellopontine angle, brain tumor, retrosigmoid craniotomy, translabyrinthine approach, posterior fossa mass, adjuvant therapy, neuro-oncology, central nervous system malignancy

## Abstract

Objective: The aim of this study was to systematically review the existing individual patient data in the literature on adult cerebellopontine angle (CPA) medulloblastoma (MB) and characterize the patient presentation, management strategies used, and oncological outcomes of this rare entity to guide future clinical practice. Methods: Following PRISMA guidelines, a systematic review was conducted by searching PubMed, EMBASE, Web of Science, and Cochrane databases from inception to 19 June 2024. Studies regarding adult patients with histologically confirmed MB radiographically confirmed to be located in the CPA were included. Clinical data were synthesized, and predictors of outcomes were evaluated. Results: Twenty-seven studies with 42 adult CPAMB patients were included. The median age was 32 years (range: 19–56). Headaches (81%), cranial neuropathy (90%), cerebellar dysfunction (79%), and nausea/vomiting (50%) were typical presenting features. The predominant histological subtype was the classic variant. Maximal safe surgical resection was performed, most commonly using a retrosigmoid approach, and 60% of cases received a gross total resection. Most patients received adjuvant treatment (93%), typically chemoradiotherapy. The recurrence rate was 11% after a median of 18 months of follow-up. Relatively high survival rates of 96%, 85%, and 85% were observed at 1, 3, and 5 years, respectively. Patients who received adjuvant therapy had significantly better recurrence and greater overall survival outcomes. Conclusions: These results support the consideration of MB in young adult patients presenting with CPA tumors with radiographical features suggestive of hypercellularity and the utilization of a management strategy of maximal safe resection plus post-operative craniospinal irradiation along with chemotherapy to optimally treat these rare patients.

## Simple Summary

This systematic review examined 42 adult cerebellopontine angle (CPA) medulloblastoma (CPAMB) patients with individual patient data from 27 studies. The median age was 32 years. Patients commonly presented with headaches (81%), cranial neuropathy (90%), cerebellar dysfunction (79%), and nausea/vomiting (50%). Maximal safe resection was pursued, and a gross total resection was performed in 60% of cases. Most patients (93%) received adjuvant therapy, typically both radiotherapy and chemotherapy. Outcomes were promising, with median survival rates of 96%, 85%, and 85% at 1, 3, and 5 years, respectively. The recurrence rate was low (11%) at a median of 18 months’ follow-up. The receipt of adjuvant therapy was significantly associated with better recurrence and survival outcomes. Medulloblastoma should be considered in the differential diagnosis of young adult patients with CPA lesions with radiographical features suggesting hypercellularity. Maximal safe resection and adjuvant craniospinal radiotherapy plus systemic therapy is an optimal management strategy.

## 1. Introduction

Medulloblastoma (MB) is a primary central nervous system (CNS) tumor found most commonly in pediatric patients. It is classically located in the midline posterior fossa, emanating from the roof of the fourth ventricle. MBs are rarely seen in adults, accounting for only 0.4–1.0% of all adult CNS tumors [1]. Medulloblastomas of the cerebellopontine angle (CPAMBs) are even rarer in adults, with fewer than 50 cases discussed in the literature [2].

The cerebellopontine angle (CPA) is a distinct anatomical region bordered superiorly by the tentorium cerebelli, posteriorly by the anterior surface of the cerebellum, inferiorly by the lower cranial nerves, anteriorly by the prepontine cistern, anterolaterally by the posterior surface of the petrous temporal bone, and medially by the pons. CPAMBs are medulloblastomas that arise in the CPA. The anatomical origin of CPAMBs is poorly understood, but they are hypothesized to originate from lateral extension through the foramina of Luschka or direct growth from the external germ layer of the cerebellum or pons [3].

Due to their rarity, the radiographical diagnosis of CPAMB is difficult in adults based on neuroimaging and clinical evaluation, as the differential diagnosis includes multiple other entities located in the CPA that are more common in adults: meningioma, vestibular schwannoma, and epidermoid cyst [4]. Similarly, most adult medulloblastomas occur in the cerebellum [5], as CPAMBs represent a rare adult MB location.

MBs are grade IV malignant embryonal tumors with both histological and molecular subtypes [6]. The histological subtypes include classic, which is the most common; desmoplastic, with a better prognosis than classic MB; and large cell/anaplastic (LCA), with a poorer prognosis [7,8]. However, the more current and clinically translatable MB stratification is based on molecular subtypes, which include *SHH*-activated (both *TP53*-wildtype and *TP53*-mutant groups) with an intermediate prognosis, *WNT*-activated with a good prognosis, and non-*WNT*/non-*SHH* (formerly group 3 and group 4 MBs) with a poorer prognosis [6].

The definitive diagnosis of CPAMB is achieved by neuropathological evaluation of surgical tumor samples [8]. Maximal safe surgical resection and adjuvant craniospinal radiation as well as chemotherapy are the current standard-of-care treatment regimen [9]. Practice is guided by management strategies used in pediatric patients, as the majority of MB patients present prior to adulthood. Due to the relative rarity of CPAMB and the more limited literature on adult patients, along with the diagnostic challenge considering common CPA lesions, this systematic review of individual patient data aims to consolidate and synthesize the published literature on adult CPAMBs to describe clinical management strategies used for these patients to inform future patient care.

## 2. Materials and Methods

### 2.1. Literature Search

The systematic review followed the recommendations of the Preferred Reporting Items for Systematic Reviews and Meta-Analyses (PRISMA). The protocol has not been registered. PubMed, EMBASE, Web of Science, and Cochrane were searched from database inception to 19 June 2024 using the Boolean full-text search [(“Medulloblastoma” OR “extra axial medulloblastoma”) AND (“Cerebellopontine Angle” OR “CPA”)]. Studies were exported to Rayyan, and duplicates were deleted.

### 2.2. Study Selection

Inclusion and exclusion criteria were defined. Articles were included if they (1) involved patients older than 18 years old with histologically confirmed CPAMB; (2) reported individual patient data, including clinical presentation, treatments used, outcome information, and follow-up data; (3) were written in English. Studies were excluded if they (1) were autopsy reports, animal studies, or studies focusing on only imaging characteristics, genetics, or histopathology; (2) were conference abstracts, literature reviews, meta-analyses, systematic reviews, perspectives, or editorials; (3) did not include or included inadequate individual patient clinical data; (4) were not written in English; (5) were not peer-reviewed.

CPA tumors were defined as those that were (1) extending exophytically into the CPA cistern, (2) located within the CPA cistern, (3) accessed through a surgical corridor into the CPA, or (4) arising from the anatomical limits of the CPA, such as the pons and/or flocculus.

Two independent reviewers (F.C. and C.J.) screened all study titles and abstracts and assessed the full texts of the articles that met the inclusion criteria. A third reviewer (K.B.) settled disagreements. Eligible papers were included, and references were screened to identify additional pertinent studies.

### 2.3. Data Extraction

One reviewer (F.C.) extracted data from each article, which was then confirmed independently by two additional reviewers (K.B. and A.F.K.). Case reports with inadequate clinical data or cohort studies lacking individual patient data were excluded. Extracted data included manuscript author, study design, sample size, patient demographics, presenting symptoms, duration of symptoms, medical comorbidities, physical exam findings, imaging modalities used during workup, tumor location, imaging characteristics of tumor, treatment approach, intraoperative details, neuropathology including immunohistochemistry (IHC) staining, adjuvant therapy, follow-up interval, clinical status at follow-up, recurrence and interventions for recurrence, overall survival, and disease-free survival.

### 2.4. Data Analysis and Quality Assessment

Descriptive statistics for the primary variables of interest were reported, including clinical characteristics, management strategies, and treatment outcomes of patients with CPAMBs. Relationships between categorical variables were assessed using Chi-square testing, with Fisher’s exact test used in instances where >20% of expected values were less than 5, in order to evaluate clinical factors that predict outcomes. Importantly, the small sample size and event numbers precluded our ability to perform Cox proportional hazards analysis to assess the temporal impact of clinical factors on outcomes over the follow-up time.

For each study, two independent authors (K.B. and G.F.) assessed the level of evidence using the 2011 Oxford Centre for Evidence-Based Medicine guidelines and the risk of bias by applying the Joanna Briggs Institute checklists for case reports and case series. Meta-analysis was not feasible, as all included studies had evidence levels IV–V, so hazard ratios (HRs) could not be deduced.

### 2.5. Statistical Analysis

SPSS V.25 (IBM Corp, Armonk, NY, USA) and Jamovi 2.3.28.0 (The Jamovi Project, open source) were utilized for all statistical analyses. Continuous variables are summarized as medians with ranges and categorical variables as frequencies with percentages. The statistical significance threshold was set at *p* < 0.05. Survival analysis, univariate analysis, and Kaplan–Meier curves were generated using Jamovi’s Survival Package.

## 3. Results

### 3.1. Study Selection and Overview

**Figure 1:**
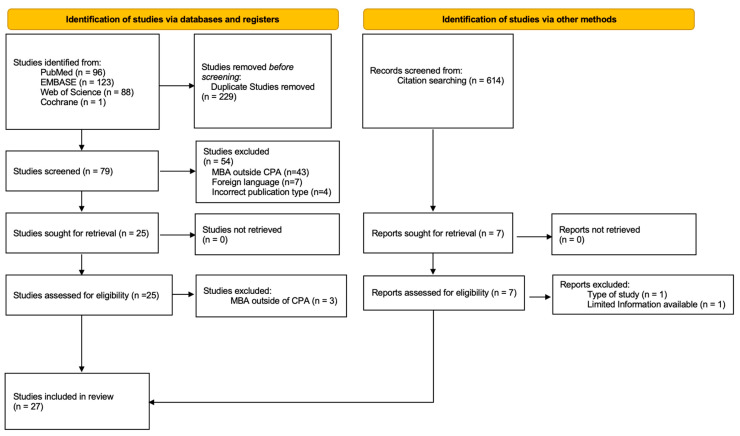
PRISMA study inclusion flowchart: this flowchart illustrates the number of studies originally identified, study exclusions, and the final inclusion of 27 studies for the review.

The search strategy yielded 308 studies (PubMed: 96; EMBASE: 123; Web of Science: 88; Cochrane: 1), of which 27 studies were included using the pre-specified study inclusion criteria (Figure 1). Three were case series (level IV evidence), and twenty-four were case reports (level V evidence). Table 1 reports the demographics, tumor location, imaging features, histology, and extent of resection for 42 patients included in these studies [2,3,4,10,11,12,13,14,15,16,17,18,19,20,21,22,23,24,25,26,27,28,29,30,31,32,33]. The critical appraisal approaches returned a low risk of bias for all included studies (Supplementary Files S1 and S2).

### 3.2. Patient Demographics and Clinical Characteristics

Table 2 outlines the composite clinical factors for the cohort. The median age was 32 years, with a range of 19 to 56 years. There was a male predominance (64%, *n* = 27). Presenting symptoms were recorded for all patients, with headaches being the most common (81%, *n* = 34), followed by nausea/vomiting (50%, *n* = 21) and gait disturbance/ataxia (40%, *n* = 17). Visual disturbances were noted in 38% (*n* = 16) of patients, hearing loss in 24% (*n* = 10), and dizziness/vertigo in 21% (*n* = 9). Tinnitus, neck discomfort, motor disturbances, and aphasia each affected 2% (*n* = 1) of the cohort. The median duration of symptoms was 3 months (range: 0.5–18 months) among the 33 patients for whom these data were available.

Physical examination signs were documented for 29 patients, revealing CN deficits in 90% (*n* = 26), cerebellar signs (i.e., ataxia, Romberg sign, gait disturbances) in 79% (*n* = 23), and papilledema in 21% (*n* = 6). CN VIII (*n* = 14, 48%), CN VII (*n* = 13, 45%), and CN VI (*n* = 6, 21%) were the most common cranial nerve deficits seen on physical exam.

### 3.3. Radiographic Findings

Table 3 outlines the neuroimaging characteristics of the tumors. Initial imaging modalities utilized by the treating teams included both computed tomography (CT) and magnetic resonance imaging (MRI) for 57% (*n* = 24) of patients, MRI alone for 36% (*n* = 15), and CT alone for 7% (*n* = 3). The majority of tumors appeared hypointense on T1 (*n* = 11, 79%), hyperintense on T2 (*n* = 10, 71%), and enhanced with gadolinium heterogeneously (*n* = 15, 68%). In cases where diffusion-weighted imaging was performed, all cases exhibited restricted diffusion. All tumors were located in the CPA, 36% (*n* = 15) without laterality specification, 33% (*n* = 14) in the right CPA, and 29% (*n* = 12) in the left CPA. A multicentric MB with bilateral CPA involvement was also included. Tumor extension outside of the CPA cistern was reported in 27 patients (64%), most frequently involving the fourth ventricle (44%, *n* = 12), tentorium cerebelli (33%, *n* = 9), and petrosal dura (26%, *n* = 7).

### 3.4. Clinical Management, Neuropathology, and Patient Outcomes

Index treatment modalities were documented for all 42 patients, with 98% (*n* = 41) undergoing microsurgical resection (Table 4). One patient (2%) was treated with radiotherapy alone. A retrosigmoid craniotomy was the most commonly used surgical approach (*n* = 28, 80% of 35 patients with these data were available). A lateral suboccipital approach was used in the remaining 20% (*n* = 7). Of the 40 patients with the available extent of resection data, a gross total resection (GTR) was reported in 60% of cases (*n* = 24). Of all included patients, complications were reported for two patients and included hemorrhage in one patient and increased hemiparesis and new-onset nystagmus in another.

The histological subtype was available for 33 patients, with classic MB being the predominant classification (56%, *n* = 19), and desmoplastic/nodular MB was observed in 36% (*n* = 12). No studies reported molecular MB subtypes.

**Figure 2:**
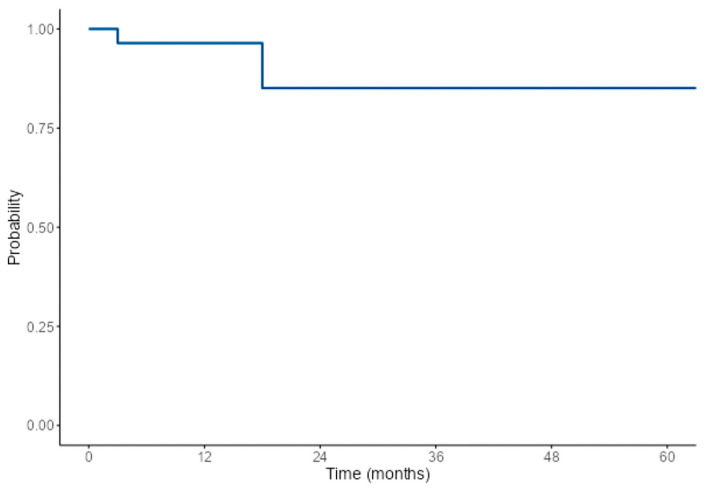
Single-arm Kaplan–Meier survival plot for overall survival: This figure portrays the unadjusted Kaplan–Meier plot showing overall survival.

**Figure 3:**
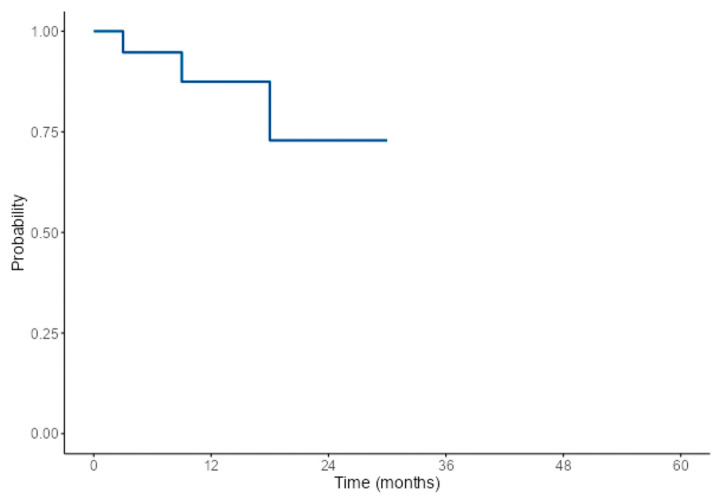
Single-arm Kaplan–Meier survival plot for recurrence-free survival: This figure portrays the unadjusted Kaplan–Meier plot showing recurrence-free survival.

Adjuvant therapy was administered to 41 patients, with 56% (*n* = 23) of these patients receiving both radiotherapy and chemotherapy, and 34% of cases (*n* = 14) were treated with radiotherapy alone. The median length of follow-up was 18 months (*n* = 34). At the last follow-up, symptoms had resolved in 50% (*n* = 12) of the 24 patients assessed, while 21% (*n* = 5) reported persistent symptoms. Recurrence was observed in 11% (*n* = 3) of the 28 patients for whom surveillance imaging results were reported. All recurrences occurred within 36 months of follow-up after initial tumor treatment, with 7% (*n* = 2) recurring within 12 months. At the last follow-up, 92% (*n* = 35) of the 38 patients with a known survival status were alive. The 1-, 3-, and 5-year overall survival rates were 96%, 85%, and 85%, respectively (Figure 2). The 1-year and 2-year recurrence-free survival rates were 87% and 73%, respectively (Figure 3).

### 3.5. Impact of Resection Status and Adjuvant Therapy on Outcomes

The proportion of patients who experienced tumor recurrences was significantly lower (*p* < 0.001) in patients who received adjuvant therapy (*n* = 1, 4%) than in those who did not (*n* = 2, 66%). There was no significant difference in recurrence-free or overall survival (OS) between patients with subtotal and gross total resection (*p* = 0.727 and *p* = 0.104, respectively).

## 4. Discussion

Adult CPA medulloblastoma is a rare subset of all medulloblastomas with few cases in the literature and with unique differential diagnostic considerations based on tumor location. Accordingly, there is a need for a systematic review to describe the management approaches used in order to guide future clinical practice. Individual patient data from 27 studies and 42 patients with CPAMBs were synthesized to provide a summary of how patients present clinically, how they are managed, and the oncological outcomes specific to this unique subset of MB.

Demographically, CPAMBs in adults present when they are in their 20s to 50s, with the median age in the 3rd decade of life. There is a slight male predominance. The relatively early age of presentation of adult CPAMBs is typical of a pediatric tumor presenting in adults and differentiates it from most adult intracranial neoplasms that tend to present in the 5th and 6th decades of life, including vestibular schwannoma and meningiomas, which are the main differential diagnoses for adult CPA tumors [34,35,36,37]. Accordingly, medulloblastomas should be considered in the differential diagnosis of young adults presenting with CPA tumors [34].

Clinical presentation is related to the anatomical location of the tumor, with adult CPAMB patients presenting due to symptomatic mass effects on adjacent cranial nerves, brainstem, and cerebellum. Headache, cranial neuropathy, cerebellar dysfunction, and nausea/vomiting were the most common presenting features. Vestibulocochlear dysfunction is a classic feature of CPA lesions due to their proximity to CN VIII, and both subjective hearing loss and CN VIII dysfunction were presenting symptoms in a subset of patients. The median duration of symptoms prior to clinical presentation was 3 months, highlighting the morbidity associated with these tumors. These presenting features align with those of pediatric CPAMBs, as expected [38].

Radiographically, CPAMBs typically appear as well-defined heterogeneously enhancing lesions on MRI with T1 hypointensity, T2 iso- or hyperintensity, diffusion-weighted imaging hyperintensity due to hypercellularity, and apparent diffusion coefficient (ADC) hypointensity [3,39,40]. However, differentiating them in adults from the significantly more common enhancing lesions in the differential diagnosis of a CPA mass, including meningiomas, ependymomas, and vestibular schwannomas, can be difficult. Comprehensive imaging is critical for operative planning and the identification of tumor extension outside of the CPA to guide maximal safe surgical resection. Overall, MB should be considered in young patients with imaging features suggestive of a hypercellular CPA lesion.

As in pediatric patients, maximal safe microsurgical resection with gross total resection, when feasible, is the standard-of-care initial treatment for most patients [41]. In this adult CPAMB cohort, 98% of the patients in our review underwent surgical resection upfront, and 60% received a GTR. CPAMBs largely presented with cranial neuropathies (90%), and tumor extension to critical surrounding neurovascular structures limited the potential for GTR in a subset of cases. In patients who underwent a STR, the authors left residual tumor to avoid new post-operative neurological deficits. Accordingly, maximal safe resection was pursued in this cohort, with GTRs when feasible and STRs accepted when safe. There was no significant difference in outcomes for patients with GTR versus STR, aligning with the MB literature and supporting the role for maximal safe resection [2]. In our experience, neuronavigation and intraoperative neuromonitoring are important surgical adjuncts for these cases to improve survival outcomes and to preserve neurologic function [41,42]. In most cases, the retrosigmoid approach was utilized. This approach provides optimal access to the CPA to facilitate maximal safe resection [43,44].

Following surgical resection, neuropathological diagnosis and MB subtyping is crucial to guide clinical management. Traditionally, MB was subtyped according to histopathology, with classic, desmoplastic, and large cell/anaplastic types. More recently, molecular subtypes of MB have been used, as they are more clinically translatable and better stratify the spectrum of disease and outcomes. The World Health Organization (WHO) criteria for MB diagnosis from 2021 categorizes them as grade IV malignant embryonal tumors and classifies them into *SHH*-activated *TP53*-wildtype, *SHH*-activated *TP53*-mutant, *WNT*-activated, and non-*WNT*/non-*SHH* subtypes [6]. The molecular MB subtypes have distinct phenotypes that impact their clinical course and outcomes [9,45]. Most adult MBs are *SHH*-activated, which are enriched for desmoplastic histology and typically located in the cerebellar hemisphere, as well as WNT-activated, which are enriched for classic histology and typically located at the midline, involving the brainstem, cerebellar peduncle, and/or the CPA [8]. The most common histological subtypes in this cohort, classic (56%) and desmoplastic (36%), align with the two most common adult MB subtypes [7]. Although *SHH*-activated desmoplastic MBs are more common in adults, selecting for CPA lesions here led to classic histology being more represented, as most CPAMBs are expected to be *WNT*-activated and, therefore, enriched for classic histology. The studies reviewed here did not describe the molecular subtypes of MBs, but they have been well described in the literature, and the neuropathological results here align with the phenotypes of the molecular groups.

Adjuvant therapy plays a crucial role in the treatment of medulloblastoma in both children and adults. In children above age three, the standard of care involves maximal safe surgical resection followed by craniospinal irradiation (CSI) and adjuvant chemotherapy consisting of combinations of vincristine, cisplatin, cyclophosphamide, and lomustine [46,47,48]. Adjuvant therapy decision-making in adults is less standardized due to the rarity of the disease and the lack of large prospective trials, but it typically follows that of pediatric patients with CSI and systemic therapy [49,50]. This was seen in our analysis, with all but three patients receiving some form of adjuvant therapy (CSI + systemic, symmetric alone, or CSI alone). Of the 24 patients who received chemotherapy, only 4 studies provided information on the specific regime (vincristine/cisplatin/cyclophosphamide, temozolomide/irinotecan, cisplatin/etoposide/cyclophosphamide, and cyclophosphamide/vincristine/lomustine). Those who received adjuvant therapy had significantly fewer recurrences; however, this finding should be taken in the context of a limited sample size. Across the cohort, 11% of patients recurred with a median follow-up of 18 months, and the 5-year survival was 85%. Symptomatically, at a median follow-up of 18 months, 50% of patients had complete symptom resolution, and 21% showed some improvement. The 1-year and 5-year survival rates were 96% and 85%, respectively. Overall, the outcomes in this cohort were good, and this review supports the use of maximal safe resection followed by adjuvant chemoradiotherapy in adult CPAMB to provide optimal patient outcomes for this aggressive tumor type.

### Limitations

This review is limited in sample size due to the rarity of the tumor of interest and the extent of individual patient data available in the literature. The literature available to review is limited to data that have been published. Potential publication bias in the literature may influence our results, for instance, if outcomes from failed therapy are underrepresented. These limitations highlight the need for collaborative, multi-center efforts to accumulate larger datasets for rare entities like CPAMB. It is important for future work to further elucidate the molecular subtype distribution of CPAMBs to guide management decisions prior to neuropathological diagnosis. This work characterizing clinical practice for adult CPAMB may lead to upcoming prospective multi-center studies to develop standard management strategies for these patients to further optimize their management. Additionally, long-term follow-up studies will be crucial for evaluating late outcomes.

## 5. Conclusions

Overall, the results of this review suggest that CPAMB should be considered in the differential diagnosis of young adult patients with CPA masses showing radiographical findings consistent with hypercellularity. This work also supports a management approach of maximal safe resection followed by adjuvant craniospinal irradiation plus systemic therapy to obtain optimal patient outcomes.

## Figures and Tables

**Table 1:** Individual patient data for included studies.

Author	Year	Study Design (Level of Evidence)	Age at Surgery	Sex	Location	Additional Radiographic Features	Histological Variant	Extent of Resection
Ebrahimzdeh et al. [10]	2022	Case Report (V)	23	Male	Right CPA	Mass effect on 4th ventricle	Large cell/anaplastic	GTR
Aqel et al. [3]	2022	Case Report (V)	43	Female	Left CPA	Mass effect on brainstem	Not Reported	GTR
Griepp et al. [4]	2022	Case Report (V)	54	Male	Left CPA	Mass effect on 4th ventricle + Dural attachment	Desmoplastic/nodular	GTR
Ali et al. [21]	2021	Case Report (V)	27	Male	Left CPA	Mass effect on 4th ventricle + dural attachment	Desmoplastic/nodular	GTR
Singh et al. [11]	2020	Case Report (V)	26	Male	Left CPA	Dural attachment	Classic	STR
Pant et al. [12]	2020	Case Report (V)	30	Female	Right CPA	Mass effect on 4th ventricle and brain stem + dural attachment	Desmoplastic/nodular	GTR
Wu et al. [2]	2020	Case Series (IV)	21	Male	CPA	Not Reported	Classic	STR
			30	Male	CPA	Not Reported	Classic	GTR
			19	Male	CPA	Not Reported	Classic	GTR
			19	Male	CPA	Not Reported	Classic	STR
			45	Male	CPA	Not Reported	Classic	STR
			42	Female	CPA	Not Reported	Classic	GTR
			34	Female	CPA	Not Reported	Classic	STR
			24	Male	CPA	Not Reported	Desmoplastic/nodular	GTR
			29	Male	CPA	Not Reported	Desmoplastic/nodular	GTR
			19	Female	CPA	Not Reported	Classic	GTR
			38	Female	CPA	Not Reported	Desmoplastic/nodular	STR
			34	Male	CPA	Not Reported	Classic	GTR
Ratha et al. [13]	2019	Case Report (V)	42	Female	Left CPA	Dural attachment	Classic	GTR
Xia et al. [22]	2019	Case Series (IV)	52	Male	CPA	Mass effect on 4th ventricle	Desmoplastic/nodular	GTR
			41	Female	CPA	Mass effect on 4th ventricle	Classic	GTR
			23	Male	CPA	Mass effect on 4th ventricle	Desmoplastic/nodular	GTR
Goudihalli et al. [23]	2018	Case Report (V)	50	Male	Right CPA	Extension into internal auditory canal and foramen magnum	Classic	STR
Batista et al. [14]	2017	Case Report (V)	25	Female	Bilateral CPA	Extension into internal auditory canal	Classic	STR
Chougule et al. [24]	2016	Case Report (V)	56	Male	Right CPA	Not Reported	Not Reported	GTR
McLaughlin et al. [15]	2014	Case Report (V)	26	Female	Right CPA	Mass effect on brainstem	Medullomyoblastoma	STR
Bahrami et al. [25]	2014	Case Report (V)	23	Male	Right CPA	Not Reported	Desmoplastic/nodular	GTR
Spina et al. [16]	2013	Case Series (IV)	22	Male	Left CPA	Dural attachment	Classic	GTR
			26	Female	Right CPA	Mass effect on brainstem	Classic	GTR
Ciccarino et al. [26]	2012	Case Report (V)	31	Male	Left CPA	Not Reported	Desmoplastic/nodular	GTR
Dalgic et al. [27]	2011	Case Report (V)	34	Male	Left CPA	Mass effect on 4th ventricle + dural attachment	Classic	STR
Behbahani et al. [28]	2011	Case Report (V)	30	Female	Right CPA	Dural attachment	Not Reported	NA
Yoshimura et al. [17]	2009	Case Report (V)	25	Female	Right CPA	No	Classic	STR
Furtado et al. [29]	2009	Case Report (V)	32	Male	Right CPA	Mass effect + dural attachment	Classic	GTR
Fallah et al. [30]	2009	Case Report (V)	47	Male	Right CPA	Dural attachment	Not Reported	Not Reported
Magliulo et al. [18]	2005	Case Report (V)	28	Male	Left CPA	Extension into internal auditory canal	Not Reported	GTR
Akay et al. [31]	2003	Case Report (V)	21	Male	Left CPA	Mass effect at 4th ventricle + dural attachment	Not Reported	STR
Kumar et al. [32]	2001	Case Series (IV)	20	Female	Right CPA	Mass effect on brainstem	Desmoplastic/nodular	GTR
			24	Male	Right CPA	Extension into cerebellum	Not Reported	STR
Mehta et al. [19]	1998	Case Report (V)	40	Male	Right CPA	Mass effect on 4th ventricle	Desmoplastic/nodular	STR
Yamada et al. [20]	1993	Case Report (V)	19	Female	Left CPA	Mass effect on 4th ventricle	Not Reported	STR
House et al. [33]	1985	Case Series (IV)	46	Male	Left CPA	Mass effect on 4th ventricle + Extension into internal auditory canal	Not Reported	STR

Abbreviations: CPA, cerebellopontine angle; GTR, gross total resection; STR, subtotal resection; NA, not applicable.

**Table 2:** Summary of patient clinical factors.

	*n* or Median	% or Range
Demographics/clinical symptoms (*n* = 42)		
Age (years)	32	19–56
Gender (male)	27	64
Presenting symptoms (*n* = 42)		
Headache	34	81
Nausea/vomiting	21	50
Gait difficulty/ataxia	17	40
Visual disturbance	16	38
Hearing loss	10	24
Dizziness/vertigo	9	21
Tinnitus	5	12
Neck discomfort	1	2
Motor disturbance	1	2
Aphasia	1	2
Duration of symptoms (months)	3	0.5–18
Presenting signs (*n* = 29)		
Cranial neuropathy	26	90
CN VIII	14	48
CN VII	13	45
CN VI	6	21
CN V	5	17
CN IX	5	17
CN X	5	17
CN XI	2	7
CN XII	1	3
Cerebellar signs	23	79
Papilledema	6	21
Weakness	3	10
Hyperreflexia	1	3
Visual deficits	1	3

Abbreviations: CN, cranial nerve.

**Table 3:** Radiographic tumor characteristics.

	*n* or Median	% or Range
Initial imaging modality (*n* = 42)		
CT and MRI	24	57
MRI only	15	36
CT only	3	7
Relative Signal Intensity T1 (*n* = 14)		
Hypointense	11	79
Isointense	2	14
Mixed	1	7
Relative Signal Intensity T2 (*n* = 14)		
Hyperintense	10	71
Mixed	3	21
Isointense	1	7
Contrast Enhancement (*n* = 22)		
Heterogeneous	15	68
Homogeneous	7	32
Diffusion-weighted imaging results (*n* = 6)		
Restricted diffusion	6	100
Tumor Location (*n* = 42)		
CPA, side not specified	15	36
Right CPA	14	33
Left CPA	12	29
Bilateral CPA	1	2
Additional Radiographic Features (*n* = 27) *		
4th ventricle extension	12	44
Tentorium cerebelli attachment	9	33
Petrosal dura attachment	7	26
Cystic component	7	26
Brainstem extension	6	22
Internal auditory canal extension	4	15
Cerebellum extension	2	7
Foramen magnum extension	1	4
CN involvement	1	4

* Patients may fit multiple categories. Abbreviations: CN, cranial nerve; CPA, cerebellopontine angle; CT, computerized tomography; MB, medulloblastoma; MRI, magnetic resonance imaging.

**Table 4:** Summary of clinical management strategies utilized, histopathology, and patient outcomes.

	*n* or Median	% or Range
Index treatment modality (*n* = 42)		
Microsurgical resection	41	98
Radiotherapy	1	2
Surgical approach (*n* = 35)		
Retrosigmoid craniotomy	28	80
Lateral suboccipital craniotomy	7	20
Extent of resection (*n* = 40)		
Gross total resection	24	60
Subtotal resection	16	40
Histological subtype (*n* = 33)		
Classic	19	56
Desmoplastic/nodular	12	36
Large cell/anaplastic	1	3
Medullomyoblastoma	1	3
Adjuvant therapy (*n* = 41)		
Radiotherapy and chemotherapy	23	56
Radiotherapy alone	14	34
Chemotherapy alone	1	2
None	3	7
Months to last follow-up (*n* = 34)	18	1–135
Symptom status at last follow-up (*n* = 24)		
Resolved	12	50
Improved	5	21
Worsened	2	8
Unchanged	5	21
Recurrence (*n* = 28)		
No	25	89
Yes	3	11
Survival status at last follow-up (*n* = 38)		
Alive	35	92
Dead	3	8

## Data Availability

No new data were created or analyzed in this study.

## Supplementary Materials

The following supporting information can be downloaded at https://www.mdpi.com/article/10.3390/cancers16244242/s1: File S1: Joanna Briggs Institute Checklist for Case Series; File S2: Joanna Briggs Institute Checklist for Case Reports.
