# Supplementary material for: Adult Cerebellopontine Angle Medulloblastoma: A Systematic Review of Clinical Features, Management Approaches, and Patient Outcomes
Source: Cancers (Basel). 2024 Dec 20;16(24):4242. doi: 10.3390/cancers16244242 (PMC11674105; doi:10.3390/cancers16244242)
Supplement: Supplementary file 1 [file cancers-16-04242-s001.zip › Supplementary File 2.docx]

**S2**: Joanna Briggs Institute Checklist for Case Reports

| **Joanna Briggs Institute Checklist for Case Reports** | | | | | | | | | |
| --- | --- | --- | --- | --- | --- | --- | --- | --- | --- |
| **Author** | **Year** | **1** | **2** | **3** | **4** | **5** | **6** | **7** | **8** |
| Ebrahimzdeh et al. | 2022 | Yes | Yes | Yes | Yes | Yes | Yes | Yes | Yes |
| Aqel et al. | 2022 | Yes | Yes | Yes | Yes | Yes | Yes | Yes | Yes |
| Griepp et al. | 2022 | Yes | Yes | Yes | Yes | Yes | Yes | Yes | Yes |
| Ali et al. | 2021 | Yes | Yes | Yes | Yes | Yes | Yes | Yes | Yes |
| Singh et al. | 2020 | Yes | Yes | Yes | Yes | Yes | Yes | Yes | Yes |
| Pant et al. | 2020 | Yes | Yes | Yes | Yes | No | Yes | Yes | Yes |
| Wu et al. | 2020 | Yes | No | Yes | Yes | Yes | Yes | Yes | Yes |
| Ratha et al. | 2019 | Yes | Yes | Yes | Yes | Yes | Yes | Yes | Yes |
| Xia et al. | 2019 | Yes | Yes | Yes | Yes | Yes | Yes | Yes | Yes |
| Goudihalli et al. | 2018 | Yes | Yes | Yes | Yes | Yes | Yes | Yes | Yes |
| Batista et al. | 2017 | Yes | Yes | Yes | Yes | Yes | Yes | Yes | Yes |
| Chougule et al. | 2016 | Yes | No | Yes | Yes | Yes | No | No | Yes |
| McLaughlin et al. | 2014 | Yes | Yes | Yes | Yes | Yes | Yes | Yes | Yes |
| Bahrami et al. | 2014 | Yes | Yes | Yes | Yes | Yes | Yes | Yes | Yes |
| Spina et al. | 2013 | Yes | Yes | Yes | Yes | Yes | Yes | Yes | Yes |
| Ciccarino et al. | 2012 | Yes | Yes | Yes | Yes | Yes | Yes | Yes | Yes |
| Dalgic et al. | 2011 | Yes | No | Yes | Yes | Yes | Yes | Yes | Yes |
| Behbahani et al. | 2011 | Yes | Yes | Yes | Yes | Yes | Yes | Yes | Yes |
| Yoshimura et al. | 2009 | Yes | Yes | Yes | Yes | Yes | Yes | Yes | Yes |
| Furtado et al. | 2009 | Yes | Yes | Yes | Yes | Yes | Yes | Yes | Yes |
| Fallah et al. | 2009 | Yes | No | Yes | Yes | No | No | No | Yes |
| Magliulo et al. | 2005 | Yes | Yes | Yes | Yes | Yes | Yes | Yes | Yes |
| Akay et al. | 2003 | Yes | Yes | Yes | Yes | Yes | Yes | Yes | Yes |
| Kumar et al. | 2001 | Yes | Yes | Yes | Yes | Yes | Yes | Yes | Yes |
| Mehta et al. | 1998 | Yes | Yes | Yes | Yes | Yes | Yes | Yes | Yes |
| Yamada et al. | 1993 | Yes | Yes | Yes | Yes | Yes | Yes | Yes | Yes |
| House et al. | 1985 | Yes | Yes | Yes | Yes | Yes | Yes | Yes | Yes |

| **Joanna Briggs Institute Checklist for Case Studies– Criteria** |
| --- |
| 1. *Were patient’s demographic characteristics clearly described?* |
| 1. *Was the patient’s history clearly described and presented as a timeline?* |
| 1. *Was the current clinical condition of the patient on presentation clearly described?* |
| 1. *Were diagnostic tests or assessment methods and the results clearly described?* |
| 1. *Was the intervention(s) or treatment procedure(s) clearly described?* |
| 1. *Was the post-intervention clinical condition clearly described?* |
| 1. *Were adverse events (harms) or unanticipated events identified and described?* |
| 1. *Does the case report provide takeaway lessons?* |
| **Responses Options: Yes, No, Unclear, Not Applicable (NA)** |
