# Supplementary material for: Adult Cerebellopontine Angle Medulloblastoma: A Systematic Review of Clinical Features, Management Approaches, and Patient Outcomes
Source: Cancers (Basel). 2024 Dec 20;16(24):4242. doi: 10.3390/cancers16244242 (PMC11674105; doi:10.3390/cancers16244242)
Supplement: Supplementary file 1 [file cancers-16-04242-s001.zip › Supplementary File 1.docx]

| **Joanna Briggs Institute Checklist for Case Series** | | | | | | | | | | | | |
| --- | --- | --- | --- | --- | --- | --- | --- | --- | --- | --- | --- | --- |
| **Study author** | **Year** | **1** | **2** | **3** | **4** | **5** | **6** | **7** | **8** | **9** | **10** | **Rating** |
| Wu et al. | 2020 | Yes | Yes | Yes | Yes | Yes | Yes | Yes | Yes | Yes | Yes | Good |
| Xia et al. | 2019 | Yes | Yes | Yes | Yes | Yes | Yes | Yes | Yes | Yes | Yes | Good |
| Spina et al. | 2013 | Yes | Yes | Yes | Yes | Yes | Yes | Yes | Yes | Yes | Yes | Good |

**S1**: Joanna Briggs Institute Checklist for Case Series

| **Joanna Briggs Institute Checklist for Case Series – Criteria** |
| --- |
| *1. Were there clear criteria for inclusion in the case series?* |
| *2. Was the condition measured in a standard, reliable way for all participants included in the case series?* |
| *3. Were valid methods used for identification of the condition for all participants included in the case series?* |
| *4. Did the case series have consecutive inclusion of participants?* |
| *5. Did the case series have complete inclusion of participants?* |
| *6. Was there clear reporting of the demographics of the participants in the study?* |
| *7. Was there clear reporting of clinical information of the participants?* |
| *8. Were the outcomes or follow up results of cases clearly reported?* |
| *9. Was there clear reporting of the presenting site(s)/clinic(s) demographic information?* |
| *10. Was statistical analysis appropriate?* |
| **Responses Options: Yes, No, Unclear, Not Applicable (NA)** |
| **Quality Rating: Poor 0 – 3; Fair 4 – 7; Good 8 – 10** |
